# Body Mass Index and Academic Achievement Among Chinese Secondary School Students: The Mediating Effect of Inhibitory Control and the Moderating Effect of Social Support

**DOI:** 10.3389/fpsyg.2022.835171

**Published:** 2022-02-21

**Authors:** Yaohui Shi, Haibo Yu, Siyu Di, Chao Ma

**Affiliations:** ^1^Normal College, Shihezi University, Shihezi, China; ^2^Center of Application of Psychological Research, Shihezi University, Shihezi, China

**Keywords:** BMI, academic achievement, inhibitory control, middle school student, social support

## Abstract

Based on Embodied Cognition Theory, Inhibitory Decline Theory, and Risk Protective Factors Model, this study verified that body mass index (BMI) affects secondary school students’ academic performance through the mechanism of inhibitory control. In addition, it was verified that the strength of this mechanism depends on the teacher, parent, and peer support received by secondary school students. By using height and weight measurements, the classic stroop task, and the social support scale, 264 secondary school students in Shanxi Province, China, were surveyed and their academic performance was collected. The results showed that students with high BMI had poorer academic performance, and inhibitory control partially mediated the effect between BMI and academic performance, with the inhibitory control mediated effect accounting for 36.68% of the total effect. Support from teachers, parents, and peers can ameliorate the negative effects of BMI on academic performance, with teacher support and parental support also ameliorating the negative effects of BMI on inhibitory control. Thus, high BMI impairs inhibitory control and thus has a negative impact on academic performance, which can be buffered by social support.

## Introduction

Body mass index (BMI), as an important indicator of an individual’s physical quality, is used to measure the total amount of body fat. And it is commonly used internationally to judge the degree of obesity, wasting and health of the human body. The World Health Organization reports that the number of obese adolescents worldwide will continue to rise in the next decade. Adolescence is a period of rapid change in body composition (location and amount of body fat) and physical fitness, and obesity at this stage may lead to a variety of physical and mental health problems in adulthood. Physiologically, obese adolescents may suffer from somatic symptoms, such as asthma, fatigue, arthralgia and dyssomnia due to excess weight (Ronghua and Xiaonan, 2006), and present a range of risk factors for chronic diseases, such as cardiovascular disease, diabetes, chronic kidney disease, cancer and musculoskeletal disorders (Afshin et al., 2017). Psychologically, overweight obesity can impede normal Self-Perception and interpersonal interactions, and most obese adolescents have mental health problems highly associated with high-risk behaviors, such as low Self-Esteem, depression, anxiety, stress, and loneliness (Paradise and Kernis, 2002). It has also been found that children with high BMI have corresponding changes in gray matter volume in brain regions, such as the fusiform gyrus, postcentral gyrus and hippocampus, which severely affects cognitive function and is detrimental to high academic achievement (Migueles et al., 2021).

In Chinese culture, academic achievement is highly valued both for students themselves and for their parents and teachers. As the Chinese proverb says, “There is gold in books” and “All things are inferior, but only reading is superior.” Academic achievement achieved in reading is considered to be a ladder to the upper class, and better academic performance means a higher social class in the future. It is precisely because academic achievement is so important in Chinese culture that teachers and families pay attention to academic achievement and ignore students’ physical quality. Students, moreover, do not have much time to focus on their physical fitness due to academic pressures, such as educational expectations, competition for higher education, and academic burdens. However, good physical fitness is essential to ensure high academic performance, where obesity may hinder students’ academic development. Therefore, it is particularly important to study the effect of BMI on academic performance.

The originality of this study is fourfold; first, it has been shown that non-overweight children achieve better academic performance than overweight children (Hermassi et al., 2021). However, criteria for overweight vary across regions and age groups, and few studies have addressed the correlation between BMI, a continuous piece of data, and academic achievement.

Second, some studies have explored why obesity negatively affects academic performance, and these studies have focused on physiological functions, with obesity leading to changes in gray matter volume in brain areas (Migueles et al., 2021) and also diminishing cardiorespiratory function (Muntaner-Mas et al., 2018), which in turn affects academic performance. It has also been demonstrated that obese children have poorer working memory and thus have difficulty achieving academic excellence (Wu et al., 2017). Whether there are more important cognitive factors (e.g., inhibitory control) that are more feasible and efficient than physical interventions require more research in the cognitive domain.

Third, previous studies have found that obese children have poorer academic performance, but there is a general phenomenon that not all overweight obese children have poorer academic performance, and which factors play an important role is currently lacking research. This study selects three groups closely related to children (teachers, parents, and peers) and explores through empirical research whether they can change the adverse effects of overweight obesity on academic performance.

Fourth, a large number of studies have examined the effects on academic achievement from cognitive and environmental aspects. This study establishes a mediated model with moderation, and adopts an interdisciplinary perspective of medicine, education, and psychology to investigate the effects of individual physiological, cognitive, and social dimensions on academic achievement, and to investigate the linkage between physical fitness, cognitive ability, social support, and education.

In the context of the global health concern of adolescent obesity and the neglect of physical fitness in schools, there is a need to explore the joint mechanisms of the effects of BMI, inhibitory control, and social support on academic performance in a study that may draw social attention to physical health and also add more interventions to improve the academic performance of obese children. Therefore, this study analyzed the effect of BMI on academic achievement and the processes and conditions underlying the effect. The first objective of the study was to analyze the correlation between BMI, inhibitory control and academic performance. The second objective analyzed the mechanisms of the effects of BMI, inhibitory control and social support on academic achievement. This study hypothesized that high BMI would have a negative impact on inhibitory control and academic achievement, high social support would improve the negative effect of high BMI on inhibitory control and academic achievement, and would also improve the negative effect of poorer inhibitory control on academic achievement.

## Literature Review

### BMI and Academic Achievement

Academic achievement is the knowledge and skills acquired by students through learning and training (McCoach et al., 2017). Embodied Cognition Theory addresses the influence of the physical structure of the body on the cognitive abilities of individuals. According to Ye et al. (2019), the way and steps in which cognitive processes are carried out are actually determined by the physical properties of the body. A view of learning based on this theory states that the body is not an irrelevant or obstructive factor in the learning process; the body is the subject of learning and physical health has an important role in shaping the mental activities of learners, such as thinking, judgment and memory (Jiayi and Haosheng, 2018). The negative correlation between BMI and academic achievement has been well verified by different scholars through cross-sectional and follow-up studies. Previous studies based on tracking data of individuals’ BMI from kindergarten to eighth grade found that adolescents with progressively and consistently higher BMI performed worse in reading and mathematics (Hsu et al., 2019). Chinese scholars investigated 1,380 fifth- and sixth-grade students and found that BMI was negatively associated with language and mathematics performance (Lv et al., 2020). Thus, BMI is an important factor influencing academic achievement.

### The Mediator Effect of Inhibitory Control

Inhibitory control is the ability of individuals to pursue cognitive representational goals by controlling their thoughts, behaviors, and attention to inhibit intrinsic dominant responses and extrinsic temptations (Diamond, 2013). Specifically, in learning activities, the ability of students to inhibit and exclude irrelevant stimuli and dominant responses that affect academic tasks with conscious involvement. More previous studies have demonstrated the relationship between BMI and inhibitory control from behavioral, neuroelectrophysiological, and physiological indices. The results of behavioral studies with cognitive deficits in obese adolescents were most consistent in executive function tests (Smith et al., 2011). There was a negative correlation between inhibitory control task performance and body weight in executive function tests, while for other components of executive function, such as working memory or cognitive flexibility, there is inconsistent results with BMI-related studies (Gunstad et al., 2008). Neuropsychological studies have found that obese subjects exhibit significantly longer N2 latency and smaller P3 wave amplitudes than normal subjects in a stroop task measuring inhibitory control (Wen and Tsai, 2020). The event-related potentials N2 and P3 are important components associated with inhibitory control. N2 is primarily associated with conflict monitoring and impulse control, with greater N2 amplitude associated with poorer inhibitory control. P3 is considered to be associated with later response decision-making and inhibitory control processes, the smaller the P3 wave amplitude, the poorer the inhibitory control (Lun, 2010). In physiological studies, overweight groups had higher leptin concentrations than the normal one. and leptin is considered to be an early indicator of cognitive impairment, with higher leptin levels associated with poorer cognitive functioning, especially in attentional inhibition control tasks (Tsai et al., 2017). Thus, compared to non-overweight secondary school students, the overweight obese have poorer inhibitory control.

As a core component of executive function, inhibitory control is an advanced cognitive function. The higher the individual’s cognitive ability, the faster and more accurate attention to key information, efficient memory coding, and produce excellent academic performance (Vock et al., 2011). The Theory of Inhibitory Depression proposed by Husher and Zucks (1988) links inhibition to working memory as well as reading understanding, arguing that the decline of inhibitory control prevents individuals to effectively suppress irrelevant information from the discourse and external environment while reading, thus reducing performance (Husher and Zucks, 1988). Titz and Karbach (2014) explored the relationship between inhibitory control and academic achievement and found that inhibitory control was correlated to some extent with verbal comprehension, writing ability, and mathematical operations. A longitudinal follow-up study of 2- to 5-year-old children also found that inhibitory control was significantly and positively associated with mathematical ability after controlling for age, maternal educational background, and children’s linguistic and lexical abilities (Espy et al., 2004). Therefore, this study hypothesized that the stronger inhibitory control of secondary school students, the better their academic performance.

Based on Embodied Cognition Theory, Inhibitory Decline Theory, and previous studies on BMI, inhibitory control, and academic performance, it can be inferred that the higher the BMI the worse the academic performance, and the deficit in inhibitory control caused by high BMI leads to poorer academic performance. Inhibitory control mediates the relationship between academic performance and BMI.

### The Moderating Effects of Social Support

It is worth noting that although overweight obesity is an important risk factor for secondary school students’ academic performance, not all overweight obese adolescents perform poorly academically, which may be due to environmental protective factors at play, of which social support is one of the important environmental protective factors. Social support is an umbrella term for the spiritual and material help individuals feel in their social life, and its ability to help them get out of trouble and better adapt to society. Based on the direct social environment of secondary school students, support from parents, teachers and peers is an important helper to help secondary school students resist risk factors. According to Risk Protective Factors Model, a classic theory that explains social support as a moderating variable (Masten, 2001), adolescent development is a dynamic interaction between protective factors (e.g., teacher support, parental support, peer support) and risk factors (poorer academic performance), in which risk factors move individuals in undesirable directions and protective factors buffer the development of such undesirable trends.

If adolescents comprehend more social support resources, their level of interpersonal trust will be higher, and the more they will be able to enhance courage and confidence in facing difficulties. Empirical studies have found that teacher support, an important attachment object for secondary school students in the school environment, can buffer the negative effects of negative factors on students’ academic performance (Luthar et al., 2000).

Higher parental support can also reduce the negative impact of negative factors on academic achievement. Longitudinal studies have shown that BMI has a negative predictive effect on academic achievement, but girls in the consistently higher BMI group did not experience poor academic achievement when they received parental support (Huang et al., 2019).

In addition, the need to belong as we enter adolescence allows peers to play an increasingly important role in the physical and mental health development of secondary school students. Some studies have shown that obese adolescents have problems with social functioning, particularly in peer relationships. Studies using photographic data have identified negative peer attitudes toward obese children, with peers often using negative terms (e.g., ugly, lazy) to describe obese children (Latner and Stunkard, 2003). Thus, teacher support, family support, and peer support may play an important role in buffering the negative effects of high BMI on inhibitory control and academic performance and the negative effects of poorer inhibitory control on academic performance.

### Research Questions

**Figure 1:**
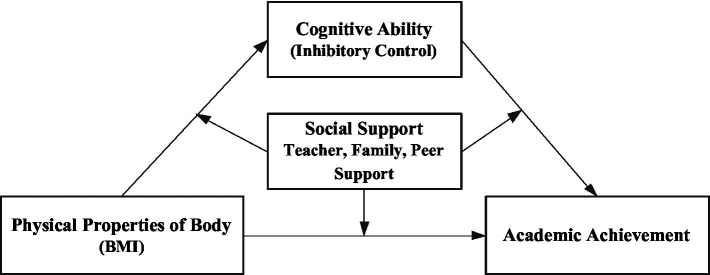
Concept framework.

The adolescent stage is a critical period for establishing lifelong healthy behaviors, but there is a lack of national research on how physical health affects educational output at this stage. Based on Embodied Cognitive Theory, Inhibitory Decline Theory, and Risk Protective Factor Model, this study attempts to investigate the effect of individual physical health (BMI) on academic performance, and the mediating role of cognitive factors (inhibitory control) and the moderating role of social support (teacher support, family support, and peer support; Figure 1) to further understand the “process” and “conditions” of the effect of BMI on academic performance of secondary school students. The purpose of this study is to further understand the “process” and “conditions” of the influence of BMI on secondary school students’ academic performance, so as to understand more objectively the influence of BMI on academic performance and its effects, and to provide an empirical basis for strengthening the concept of health investment and improving the quality education system, which is important for accelerating the implementation of the Health China Strategy.

## Materials and Methods

### Participants

Using a convenience sampling method, this survey was conducted from December 2020 to January 2021, and the sample size was calculated using correlation analysis in G Power software, assuming Effect size = 0.2, *α* = 0.05, and 1−*β* = 0.95, and 262 subjects were selected for the study according to this assumption. Because this study took continuous data of BMI to calculate the correlation with other variables, it was required that the percentage of overweight among the selected subjects was in line with the current overweight detection rate of secondary school students. According to the China Child Development Report, the rate of overweight among secondary school students in China in 2019 was 21%. In this study, secondary obese subjects were excluded, and the critical value of age-sex BMI according to the national standard (WS/T586-2018) was used as the criterion for judging overweight, and the age range of students in this study was 13.5–17.0 years old, and 264 people were finally included according to the following criteria, including 56 overweight (24 boys and 32 girls) and 208 non-overweight (100 boys and 108 girls) with a mean age of (15.00 ± 0.68) years (Table 1).

**Table 1:** BMI threshold for overweight screening.

Age (year)	Boy (kg/m^2^)	Girl (kg/m^2^)
13.5~	21.9	22.6
14.0~	22.3	22.8
14.5~	22.6	23.0
15.0~	22.9	23.2
15.5~	23.1	23.4
16.0~	23.3	23.6
16.5~	23.5	23.7
17.0~	23.7	23.8

## Measures

### Body Mass Index

In this study, height and weight were measured using the equipment and methods prescribed by national standards. According to the BMI = weight (kg)/[height (m)]^2^, height was measured in “centimeters” and recorded to one decimal place, which was converted to “meters” and retained one decimal place when calculating the BMI.

### Inhibitory Control Tasks

**Figure 2:**
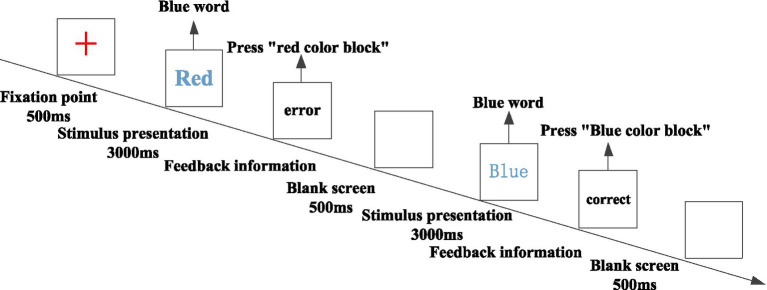
Stroop task program.

The classical stroop task was used to measure subjects’ inhibitory control, and it has been demonstrated that this paradigm can effectively measure subjects’ inhibitory control (Xuejun and Haijuan, 2018). The stimulus materials were four Chinese characters “red,” “green,” “yellow,” and “blue,” respectively written in “red,” “green,” “yellow,” and “blue” colors, which were used as “The size of each character was 1 × 1 cm. The subjects were asked to respond to the color of the presented stimulus by pressing the key, red by red block, green by green block, yellow by yellow block, and blue by blue block. The entire experiment was divided into practice and formal experiments, and the experimental procedure was the same in both phases. There were 20 practice sessions, and the total number of stimuli in the formal experiment was 160, including 40 word-color consistent stimuli and 120 inconsistent stimuli. First, a gaze point “+” appeared in the center of the screen for 500 ms, then an experimental stimulus (color word) appeared, and the program allowed a response time of 3,000 ms, and the time interval between the correct response and the next experimental stimulus was 500 ms. The subject pressed the correct key and was presented with a 1,000 ms “correct response” message on the screen. If the subject pressed a correct key, a “correct response” message was presented on the screen for 1,000 ms, and if the subject pressed an incorrect key, an “incorrect feedback” message was presented on the screen as a penalty, in order to ensure a high correct rate. The difference in response time between the inconsistent and consistent conditions was used as a measure of individual inhibitory control, and the higher the inhibitory control score, the worse the inhibitory control of the subject. Because this study focused on inhibitory control, the inhibitory control score was reverse scored as a measure of inhibitory control. After reverse scoring, the higher the inhibitory control score, the stronger the inhibitory control ability (Figure 2).

### Academic Achievement

In this study, the results of the last two examinations of senior students in a middle school in Shanxi province were collected, provided by the school’s teaching office, including nine subjects: language, mathematics, English, politics, history, geography, physics, chemistry, and biology, and the sum of the nine subject scores was used as a measure of academic achievement, and the larger the value, the higher the academic achievement of the student.

### Social Support

In this study, the scale translated and revised by Zemit compiled by Chinese scholars Yan and Xue (2006) was adopted. Two scholars adapted the Understanding Social Support Scale (MSPSS) based on the actual situation, which focused on individuals feeling the social support provided by different groups and measured the degree of individual understanding. The scale includes 12 topics, three dimensions, namely family support, friend support and teacher support, each dimension has four entries, each entry from the level 1–7 scoring method of “extreme disagree” to “extreme consent,” which is divided into the total of all scoring items, the higher the score, the higher the level of social support of individual perception. In this study, the Cronbach’s *α* coefficients of the three questionnaires were 0.84, 0.87, and 0.83.

### Procedure

Prior to the study, participants gave written informed consent in accordance with the Declaration of Helsinki. Ethical approval for this investigation was obtained from this Shihezi University review board (KJ2020-125-01).

After informed consent from the school director and the subjects, group testing was conducted in classes with two professionally trained master testers in each class. Subjects completed measures of height, weight, inhibitory control, and social support in a comfortable and quiet computer classroom (the same procedure is completed Monday through Friday from 9:30 a.m. to 10:30 a.m., temperature: 18.5 ± 0.5°C; relative humidity: 55 ± 5%). Subjects were advised to maintain their habitual dietary intake and to eat for more than 2 h before the test. Hunger level, thirst level, and eating cravings were all 0.

First, height and weight were measured using the equipment and methods specified in Chinese GB/T 26343. Second, after the main test experimenter filled in the measured height and weight on the questionnaire, they were given to the subjects to fill in other contents, including school, name, class, gender, and other basic information and comprehension of social support. In addition, the subject installed E-prime software on the computer in the school computer room, and the subject completed the test of inhibition control on the computer. Finally, the main examiner obtains the results of the last two exams through the school’s Registrar’s Office.

### Data Analysis

The statistical analyses were carried out through the IBM SPSS Statistics 22 and the modeling tool PROCESS 3.0 for SPSS (Hayes, 2018).

## Results

In this study, Self-Reported questionnaires were used to collect social support data, and results might be influenced by common method bias. Therefore, the Harman’s single-factor test was used to assess common method bias before data analysis. The results showed that eigenvalues of three unrotated factors were greater than 1, and the amount of variation explained by the first factor was 37.72%, which is much less than 40% of the critical value. Accordingly, common method bias was not significant in this study (Hao and Lirong, 2004).

### Descriptives and Correlations

Table 2 shows mean and the standard deviation of each of the variables considered in the study, and it presents the Spearman correlation coefficients among all the variables. There was significantly uncorrelated between BMI and teacher support, BMI and peer support. Other variables associations were statistically significant (*p* < 0.01).

**Table 2:** Descriptive analysis and correlation coefficients of the study variables.

	1	2	3	4	5	6	7
1. BMI	1						
2. Inhibitory control	−0.37^***^	1					
3. Social support	0.08	0.27^***^	1				
4. Teacher support	−0.02	0.22^***^	0.72^***^	1			
5. Family support	0.19^**^	0.14^*^	0.72^***^	0.22^***^	1		
6. Friend support	0.01	0.23^***^	0.78^***^	0.35^***^	0.41^***^	1	
7. Academic achievement	−0.25^***^	0.31^***^	0.53^***^	0.30^***^	0.41^***^	0.47^***^	1
Mean	20.96	-77.67	56.96	16.42	20.10	20.43	446.62
Standard deviation	3.63	63.40	11.24	5.31	4.91	4.92	60.50

* The correlation is significant at the 0.05 level.

** The correlation is significant at the 0.01 level.

*** The correlation is significant at the 0.001 level.

### Mediating Effect Analysis

According to the theory and hypothesis, the mediating effect model of the inhibitory control on the relationship between BMI and academic achievement was constructed. The mediation role of the inhibitory control between the physical fitness index and the academic performance was analyzed, using the physical fitness index as the independent variable, and using the Model 4 in the PROCESS program developed by Hayes. Stepwise regression analysis showed (Table 3) that the regression of academic achievement to BMI (*c* = −0.26, *t* = −4.21, *p* < 0.001), the regression of inhibitory control to BMI (*a* = −0.38, *t* = −6.35, *p* < 0.001), and the regression of academic achievement to BMI (*C’* = −0.16, *t* = −2.55, *p* < 0.05) and inhibitory control (*b* = 0.25, *t* = 4.06, *p* < 0.001) were all significant. Thus, inhibitory control played a partial mediating role in BMI and academic achievement.

**Table 3:** The mediating effect of the inhibitory control on the relationship between BMI and academic achievement.

Regression equation		Fit indices			Significance of regression coefficient		
Result variable	Predictor variable	*R*	*R* ^2^	*F*	*β*	SE	*t*
Academic achievement	BMI	0.25	0.06	17.76^***^	−0.26	0.06	−4.21^***^
Inhibitory control	BMI	0.37	0.13	40.29^***^	−0.38	0.06	−6.35^***^
Academic achievement	BMI	0.35	0.12	17.66^***^	−0.16	0.06	−2.55^*^
	Inhibitory control				0.25	0.06	4.06^***^

* The correlation is significant at the 0.05 level.

*** The correlation is significant at the 0.001 level.

In order to validate the significance of the mediating effect, non-parametric Bootstrap method was also used (repeat sampling 5,000 times). As shown in Table 4, the results showed that the 95% confidence interval corresponding to each path did not contain 0, indicating that the total effect, direct effect and indirect effect were statistically significant (*p* < 0.05). Thus, the mediating effect of the inhibitory control on the relationship between BMI and academic achievement was statistically significant. The mediation effect value was −0.10, accounting for 38.46% (−0.10)/(−0.26) of the total effect.

**Table 4:** Total effect, direct effect and mediating effect.

	Mediation path	Standardized effect value	Bootstrap standard error	95% CI	Effect size
Total effect	Path1	−0.26^***^	0.06	[−0.38, −0.14]	
Direct effect	Path2	−0.16^*^	0.06	[−0.29, −0.04]	61.54%
Mediating effect	Path3	−0.10^***^	0.03	[−0.15, −0.04]	38.46%

*Path1 BMI→Academic achievement, Path2 BMI→Academic achievement, Path3 BMI→Inhibitory control→Academic achievement.*

* The correlation is significant at the 0.05 level.

*** The correlation is significant at the 0.001 level.

### Moderated Mediation Effect Analysis

The moderating effects of teacher support, family support and peer support in the mediation model were analyzed by using the model 59 in PROCESS.

#### The Moderating Effect of the Teacher Support

**Figure 3:**
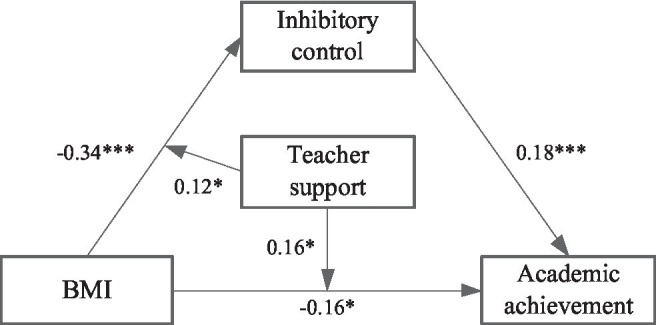
A moderated mediation model of the teacher support. *The correlation is significant at the 0.05 level. ***The correlation is significant at the 0.001 level.

In the first step, a model (Figure 3) is constructed to analyze the moderating role of teacher support in mediating effect. As shown in Table 5, the results showed that BMI had a significant effect on inhibitory control (*β* = −0.34, *t* = −5.87, *p* < 0.001), and the effect of BMI × teacher support to inhibitory control was significant (*β* = 0.12, *t* = 2.23, *p* < 0.05), indicating that the relationship between BMI and inhibitory control was moderated by teacher support. BMI had a significant effect on academic achievement (*β* = −0.16, *t* = −2.69, *p* < 0.01), and the effect of BMI × teacher support to academic achievement was significant (*β* = 0.16, *t* = 2.71, *p* < 0.01), indicating that the relationship between BMI and academic achievement was moderated by teacher support. Inhibitory control had a significant effect on academic achievement (*β* = 0.18, *t* = 2.88, *p* < 0.01), but the effect of inhibitory control × teacher support to academic achievement was not significant (*β* = 0.08, *t* = 1.34, *p* > 0.05), indicating that the relationship between inhibitory control and academic achievement was not moderated by teacher support.

**Table 5:** The moderating effect of the teacher support on the mediating effect.

Regression equation		Fit indices			Significance of regression coefficient	
Result variable	Predictor variable	*R*	*R* ^2^	*F*	*β*	*t*
Inhibitory control	BMI	0.44	0.20	21.08^***^	−0.34	−5.87^***^
	Teacher support				0.21	3.84^**^
	BMI × Teacher support				0.12	2.23^*^
Academic achievement	BMI	0.45	0.20	13.14^***^	−0.17	−2.69^**^
	Teacher support				0.26	4.58^***^
	BMI × Teacher support				0.16	2.71^**^
	Inhibitory control				0.18	2.88^**^
	Inhibitory control × Teacher support				0.08	1.34

* The correlation is significant at the 0.05 level.

** The correlation is significant at the 0.01 level.

*** The correlation is significant at the 0.001 level.

**Figure 4:**
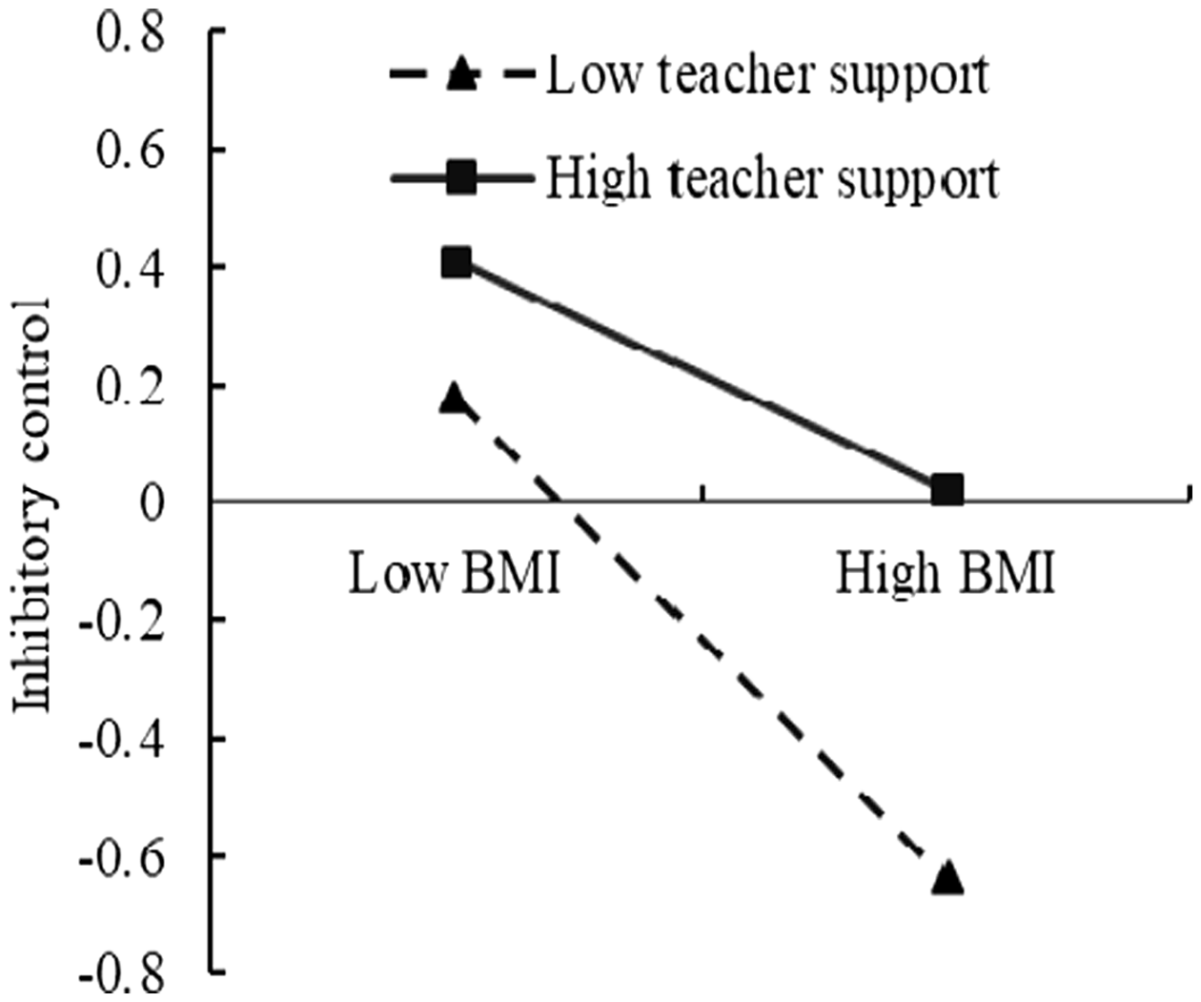
The moderating effect of the teacher support on the relationship between BMI and inhibitory control.

**Figure 5:**
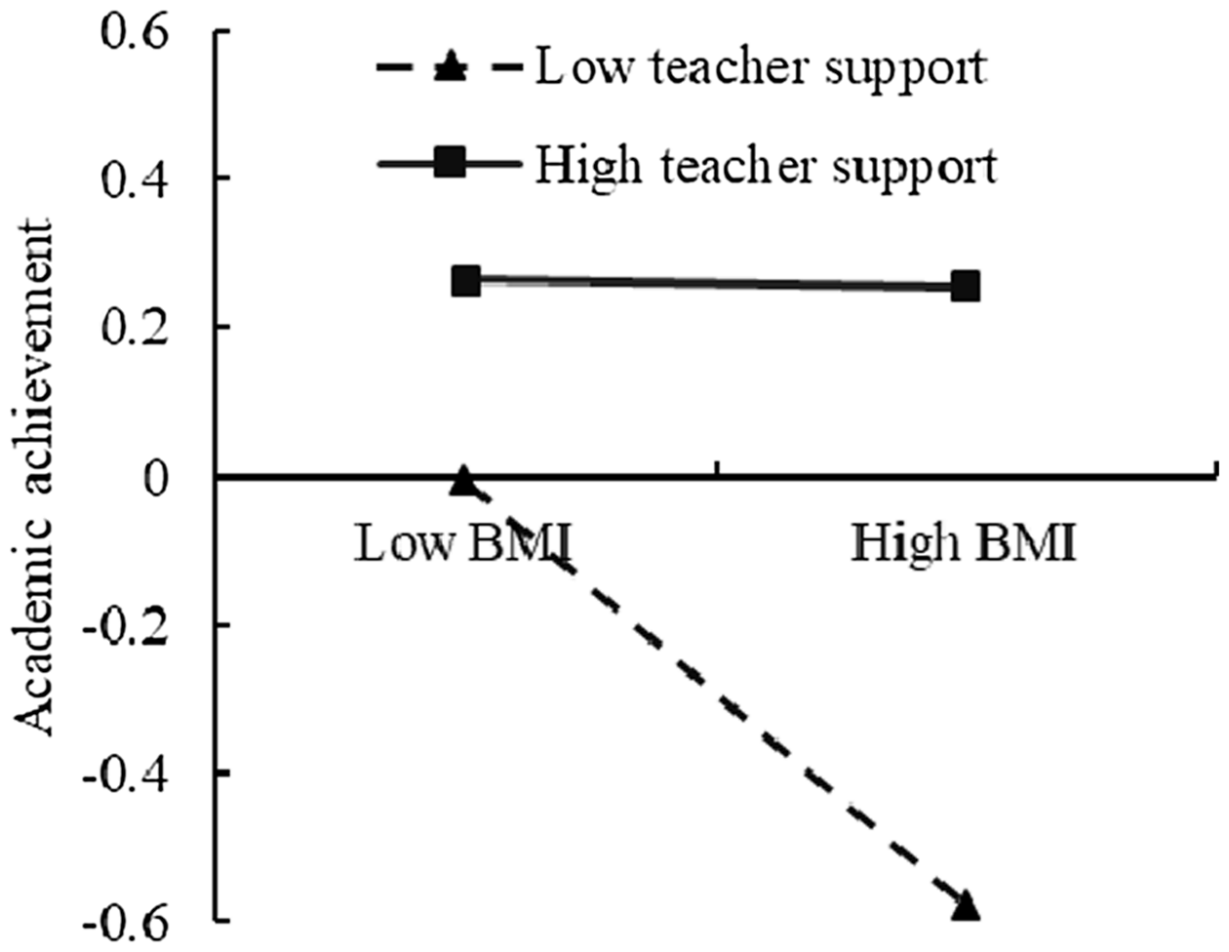
The moderating effect of the teacher support on the relationship between BMI and academic achievement.

To further analyze the moderating effect of the teacher support, the teacher support was divided into the high and low groups, according to the principle of standard deviation, and a simple slope test was performed (Figures 4, 5). The results found that for individuals with low score of teacher support, BMI could significantly predict inhibitory control (*β* = −0.46, *t* = −6.60, *p* < 0.001). For individuals with high score of teacher support, prediction effect of BMI to inhibitory control decreased (*β* = −0.23, *t* = −2.62, *p* < 0.01). For individuals with low score of teacher support, BMI could significantly predict academic achievement (*β* = −0.32, *t* = −4.04, *p* < 0.01). For individuals with high score of teacher support, prediction of BMI to academic achievement was not significant (*β* = −0.01, *t* = −0.15, *p >* 0.05).

#### The Moderating Effect of the Family Support

**Figure 6:**
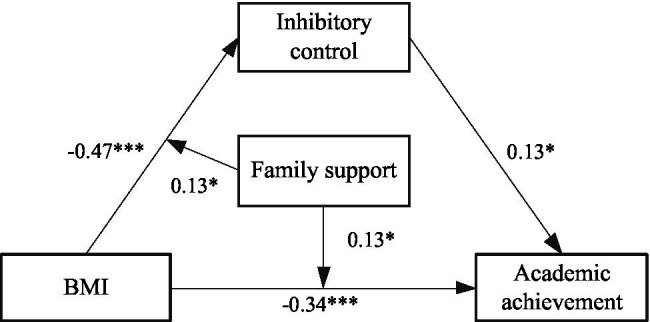
A moderated mediation model of the family support. *The correlation is significant at the 0.05 level. ***The correlation is significant at the 0.001 level.

Next, a model (Figure 6) is constructed to analyze the moderating role of family support in mediating effect. As shown in Table 6, the results showed that BMI had a significant effect on inhibitory control (*β* = −0.47, *t* = −7.49, *p* < 0.001), and the effect of BMI × family support to inhibitory control was significant (*β* = 0.13, *t* = 2.19, *p* < 0.05), indicating that the relationship between BMI and inhibitory control was moderated by family support. BMI had a significant effect on academic achievement (*β* = −0.34, *t* = −5.36, *p* < 0.001), and the effect of BMI × family support to academic achievement was significant (*β* = 0.12, *t* = 2.05, *p* < 0.05), indicating that the relationship between BMI and inhibitory control was moderated by family support. Inhibitory control had a significant effect on academic achievement (*β* = 0.13, *t* = 2.27, *p* < 0.05), but the effect of inhibitory control× family support to academic achievement was not significant (*β* = −0.02, *t* = −0.32, *p >* 0.05), indicating that the relationship between inhibitory control and academic achievement was not moderated by family support.

**Table 6:** The moderating effect of the family support on the mediating effect.

Regression equation		Fit indices			Significance of regression coefficient	
Result variable	Predictor variable	*R*	*R* ^2^	*F*	*β*	*t*
Inhibitory control	BMI	0.44	0.19	20.81^***^	−0.47	−7.49^***^
	Family support				0.23	4.01^***^
	BMI × Family support				0.13	2.19^*^
Academic achievement	BMI	0.56	0.31	23.47^***^	−0.34	−5.36^***^
	Family support				0.46	8.36^***^
	BMI × Family support				0.12	2.05^*^
	Inhibitory control				0.13	2.27^*^
	Inhibitory control × Family support				−0.02	−0.32

* The correlation is significant at the 0.05 level.

*** The correlation is significant at the 0.001 level.

**Figure 7:**
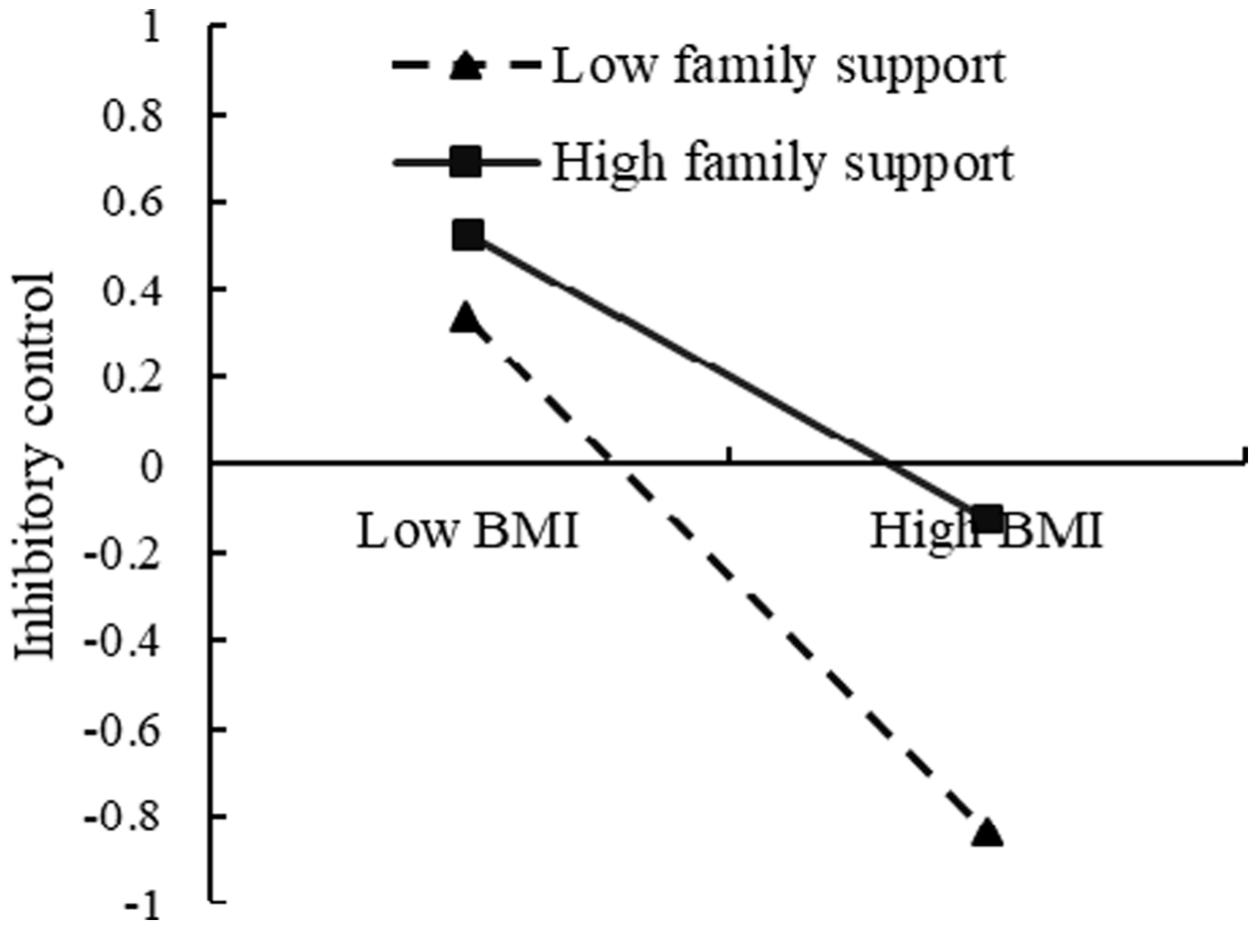
The moderating effect of the family support on the relationship between BMI and inhibitory control.

**Figure 8:**
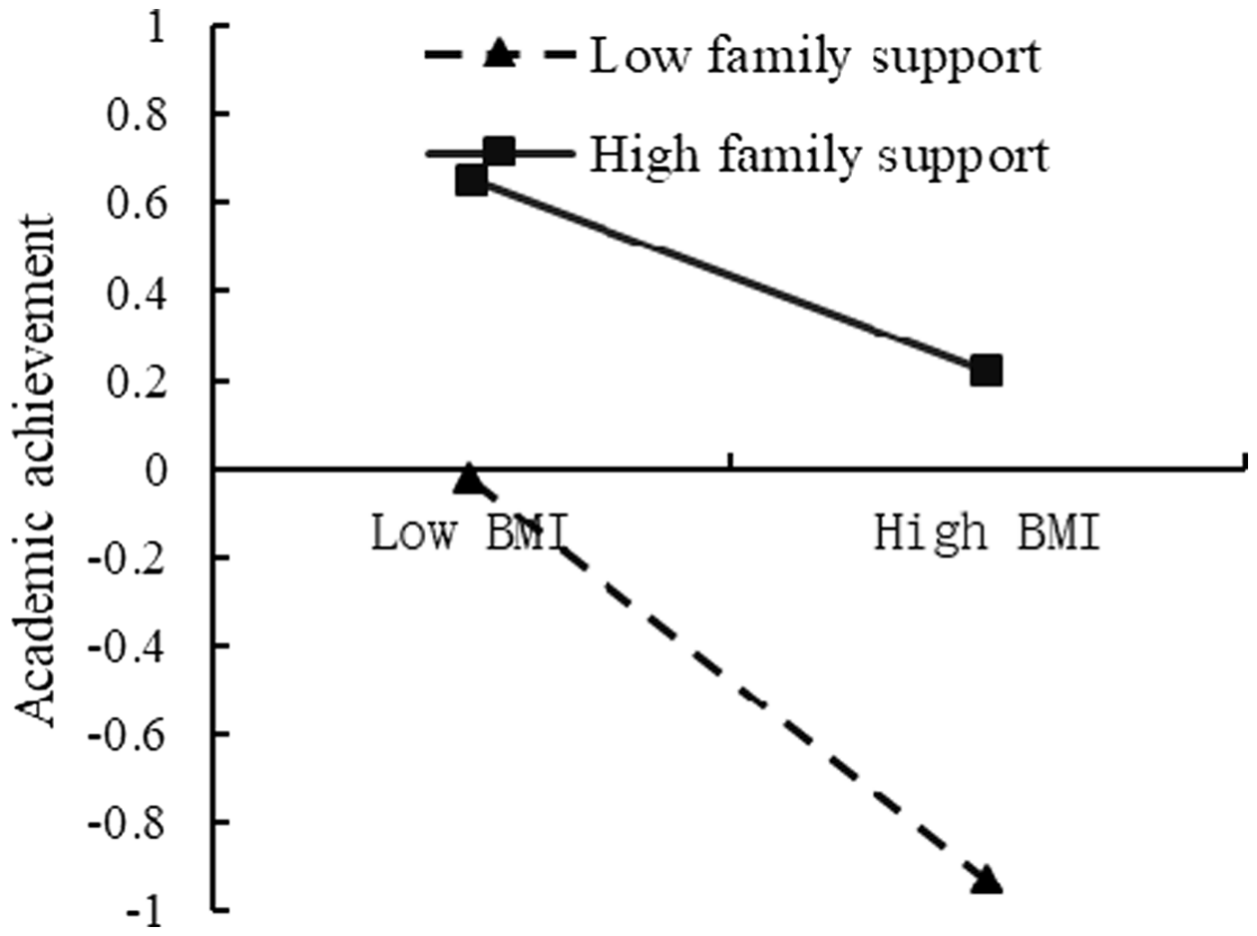
The moderating effect of the family support on the relationship between BMI and academic achievement.

To further analyze the moderating effect of the family support, the family support was divided into the high and low groups, according to the principle of standard deviation, and a simple slope test was performed (Figures 7, 8). The results found that for individuals with low score of family support, BMI could significantly predict inhibitory control (*β* = −0.60, *t* = −5.93, *p* < 0.001). For individuals with high score of family support, prediction effect of BMI to inhibitory control decreased (*β* = −0.33, *t* = −4.74, *p <* 0.001). For individuals with low score of family support, BMI could significantly predict academic achievement (*β* = −0.46, *t* = −4.55, *p* < 0.001). For individuals with low score of family support, prediction effect of BMI to academic achievement decreased (*β* = −0.22, *t* = −3.25, *p* < 0.01).

#### The Moderating Effect of the Peer Support

**Figure 9:**
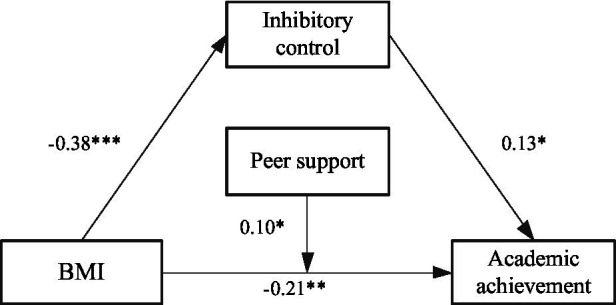
A moderated mediation model of the peer support. *The correlation is significant at the 0.05 level. **The correlation is significant at the 0.01 level. ***The correlation is significant at the 0.001 level.

In the third step, a model (Figure 9) is constructed to analyze the moderating role of peer support in mediating effect. As shown in Table 7, the results showed that BMI had a significant effect on inhibitory control (*β* = −0.38, *t* = −6.56, *p* < 0.001), and the effect of BMI × peer support to inhibitory control was not significant (*β* = 0.02, *t* = 0.49, *p >* 0.05), indicating that the relationship between BMI and inhibitory control was not moderated by peer support. BMI had a significant effect on academic achievement (*β* = −0.21, *t* = −3.67, *p* < 0.01), and the effect of BMI × peer support to academic achievement was significant (*β* = 0.10, *t* = 2.03, *p* < 0.05), indicating that the relationship between BMI and academic achievement was moderated by peer support. Inhibitory control had a significant effect on academic achievement (*β* = 0.13, *t* = 2.26, *p* < 0.05), but the effect of inhibitory control × peer support to academic achievement was not significant (*β* = −0.01, *t* = −0.17, *p >* 0.05), indicating that the relationship between inhibitory control and academic achievement was not moderated by peer support.

**Table 7:** The moderating effect of the peer support on the mediating effect.

Regression equation		Fit indices			Significance of regression coefficient	
Result variable	Predictor variable	*R*	*R* ^2^	*F*	*β*	*t*
Inhibitory control	BMI	0.44	0.19	20.19^***^	−0.38	−6.56^***^
	Peer support				0.23	3.98^**^
	BMI × Peer support				0.02	0.49
Academic achievement	BMI	0.56	0.32	24.12^***^	−0.21	−3.67^**^
	Peer support				0.42	7.54^***^
	BMI × Peer support				0.10	2.03^*^
	Inhibitory control				0.13	2.26^*^
	Inhibitory control × Peer support				−0.01	−0.17

* The correlation is significant at the 0.05 level.

** The correlation is significant at the 0.01 level.

*** The correlation is significant at the 0.001 level.

**Figure 10:**
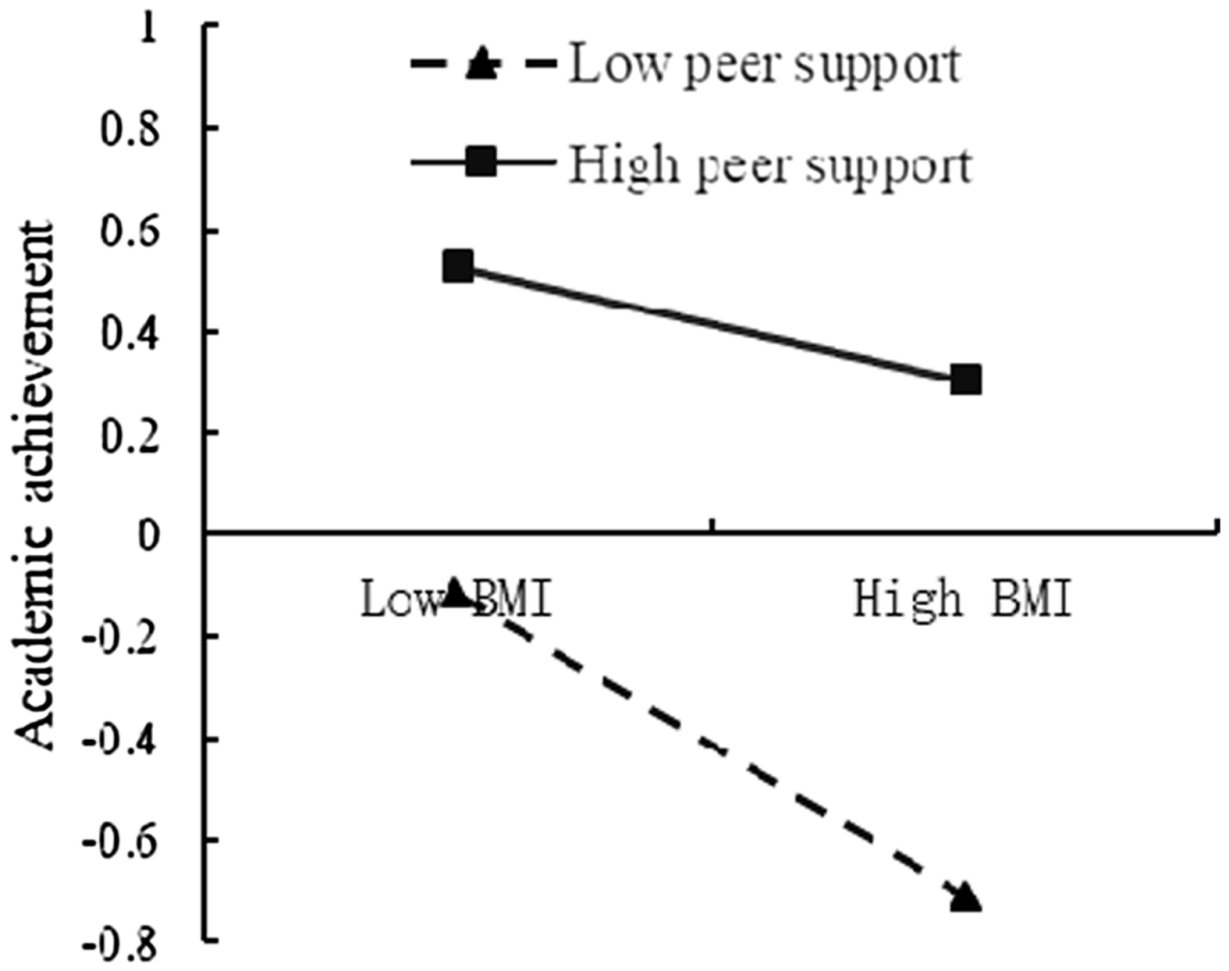
The moderating effect of the peer support on the relationship between BMI and academic achievement.

To further analyze the moderating effect of the peer support, the peer support was divided into the high and low groups, according to the principle of standard deviation, and a simple slope test was performed (Figure 10). The results found that for individuals with low score of peer support, BMI could significantly predict academic achievement (*β* = −0.30, *t* = −4.09, *p* < 0.001). For individuals with high score of peer support, prediction of BMI to academic achievement was not significant (*β* = −0.12, *t* = −1.57, *p* > 0.05).

## Discussion

There are three important findings from this study. First, the findings suggest that high BMI secondary school students are associated with poor academic performance. Second, high BMI secondary school students’ poor performance in school was partly due to their poor inhibitory control. Third, some high BMI academic performance was not poor because of high social support from teachers, parents, and peers. Similarly, some high BMI secondary school students’ inhibitory control is not poor because of high social support from teachers and parents.

### Influence of BMI on Academic Achievement

Consistent with the research hypothesis and previous studies, the present study found that high BMI negatively affects academic achievement. Overweight adolescents experience poorer school performance, such as lower math and reading scores, IQ scores, GPA, and absenteeism (Finn et al., 2018). Meanwhile, the results have been argued across cultures, with a review of nine studies from the United States, Western Europe, South America, and Asia showing a significant negative association between BMI and academic performance (Taras and Potts-Datema, 2005). The present study adds to the research related to this area of obesity and academic performance in Chinese adolescents. Conversely, some studies have found that obesity does not affect academic performance (Barrigas and Fragoso, 2012). The measurement methods and standards of obesity, the measurement of academic performance, and the maturity of the subjects may explain this difference. In contrast to previous studies that examined differences in academic achievement between overweight and non-overweight groups, the use of BMI continuous data in this study avoids differences in results due to different criteria for overweight. In this study, the correlation between BMI and academic achievement was small (*r* = −0.252), a result that is consistent with the most recent meta-analysis, which showed a weak negative correlation between BMI and academic achievement (−0.067 < *r* < 0.155), and it was noted in this study that the magnitude of the correlation coefficient was influenced by grade level, compared to elementary and middle school students, the correlation coefficient was the largest for high school students (*r* = −0.184; He et al., 2019). Therefore, the correlation in our study is reasonable because our sample was selected from high school students.

### The Mediating Effect of Inhibition Control

Consistent with the research hypothesis, the mediation analysis in this study indicated that BMI was predictive of their academic performance through inhibition control. A systematic review also pointed out the relationship between obesity, cognitive function, and academic achievement, with obese individuals having poorer executive function, attention, and visual space compared to those of normal weight at the same age, and these factors were significant positive predictors of academic achievement (Liang et al., 2014). A meta-analysis that included 4,904 subjects found that overweight obese subjects had significantly impaired inhibitory control compared to normal weight (Yang et al., 2017). Exploring the reasons for the poor inhibitory control, on the one hand, it may be that overweight obese subjects are unable to restrain themselves when confronted with food and may have binge eating, and the negative effects of this behavior may occur in various ways, one of which is impairedt inhibitory control (Balodis et al., 2013). On the other hand obese adolescents have a lower maturation of the right frontal neurological area associated with inhibitory control and a lower level of prefrontal cortex activity, leading to an inability to rapidly activate conflict monitoring abilities (Alatorre-Cruz et al., 2021).

Inhibitory control as a higher cognitive ability has a positive predictive effect on academic performance. Consistent with meta-analytic findings, the present study also confirmed that inhibitory control is a significant predictor variable of academic ability, and that the components of inhibitory control focus attention and inhibit impulsive responses are fundamental to the completion of academic tasks and learning (Cortés Pascual et al., 2019). Compared to previous studies that found obesity to adversely affect academic performance through changes in cardiorespiratory function and brain area volume, the mediating variable inhibitory control in this study was more feasible for intervention. A computer-based intervention study showed a significant increase in students’ academic performance following a suppression control “Stop & Think” intervention (Wilkinson et al., 2020).

### The Moderating Role of Social Support in the Relationship Between BMI and Academic Achievement

This study found that teacher support, family support, and peer support all mitigate the negative effects of high BMI on academic achievement. Previous studies did not consider the role of social support in the relationship between BMI and academic achievement. When social support was included as a variable, social support determined the strength of the relationship between BMI and academic achievement.

First, there was no significant difference in academic achievement between overweight obese and non-overweight adolescents when receiving high teacher support. This emphasizes the importance of teacher support, When overweight obese adolescents perceive acceptance by their teachers, it stimulates intrinsic motivation to learn and increases classroom participation, which in turn drives academic development (Saeed et al., 2021). This is consistent with previous research findings that teacher support significantly and positively predicts students’ academic achievement, especially among disadvantaged groups, which are more sensitive to support from teachers. More emotional support from teachers to disadvantaged students can bring good emotional experiences for students, which can make them feel psychologically warm, which in turn stimulates strong motivation, high attention span, and contributes to superior academic achievement (Liu et al., 2019).

Second, family support can attenuate the negative effects of high BMI on academic performance. This is consistent with previous research, which has found that the negative predictive effect of overweight obesity on academic achievement in girls has been found to be insignificant possibly due to the support girls receive from their families (Black et al., 2015). Also, this result is consistent with Social Learning Theory, which individuals emphasize the importance of imitating objects and their characteristics to motivate specific behaviors (Chavis, 2011). Children often observe and imitate their parents as role models, and their parents’ correct attitude toward learning is an indirect moral support, and this support promotes the occurrence of positive learning behaviors.

Finally, there is no significant difference in academic achievement between overweight obese adolescents and non-overweight secondary school students when receiving high peer support, which highlights the importance of peer support for overweight obese secondary school students as reflected in high or low academic achievement. Overweight obese children are more likely to be victims and aggressors of bullying than their normal-weight peers, and studies have found that obese children are subjected to verbal abuse, teasing, and kicking by their peers (Latner and Stunkard, 2003). Lower peer acceptance often predicts lower social support, which severely affects the academic performance of overweight obese children (Lv et al., 2020). In contrast, positive peer relationships are often associated with more material support (e.g., shared learning resources) and emotional support (e.g., mitigating the negative effects of academic stress) from peers, which contributes to superior academic performance (Gallardo et al., 2016).

### The Moderating Role of Social Support in the Relationship Between BMI and Inhibition Control

The present study found that in addition to peer support, teacher support and family support can mitigate the adverse effects of high BMI on inhibitory control.

First, teacher support can weaken the negative effect of high BMI on inhibitory control. This result is consistent with the teacher Expectancy Effect Theory, which states that when teachers invest more expectations and positive emotions in students, the more likely students are to develop in the direction teachers expect them to, and teachers expect students to have higher inhibitory control. Previous research has also found that when there is a lack of teacher support, it leads to poor Self-Regulation, and inhibitory control is a cognitive manifestation of Self-Regulation (Curby et al., 2013).

Second, family support can attenuate the negative effects of high BMI on inhibitory control. This result is consistent with Social Control Theory, which suggests that the social environment restricts people from engaging in socially detrimental behaviors, that parental support in the home environment is a constructive way to substantially increase inhibitory control, and that enthusiastic parental support will enhance children’s ability to control impulses, which will contribute to the development of their inhibitory control (Wilder, 2014). Thus, healthy parent–child relationships allow adolescents to explore the world fully, gain more energy, and improve the effects of adverse factors on inhibitory control.

Finally, peer support does not diminish the negative effects of high BMI on inhibitory control. There is a proverb in Chinese culture that “those who are close to vermilion are red, those who are close to ink are black,” which means that we need to choose our friends carefully, as good friends will fill our lives with positive energy, while bad friends will lead us to the abyss. As in social learning theory, when individuals observe and imitate bad behavior habits, such as get poorer inhibitory control, fail to view peer support correctly and identify “same absenteeism” as high peer support from companionship, this support can instead hinder inhibitory control development.

### Social Support Has No Moderating Effect on the Relationship Between Inhibitory Control and Academic Achievement

This study found that teacher support, family support, and peer support did not mitigate the negative effects of low inhibitory control on academic achievement. This is inconsistent with the research hypothesis, and the results of this study reveal that not all negative effects of adverse factors on academic achievement can be ameliorated by social support, highlighting the importance of inhibitory control as a relatively stable cognitive ability on academic achievement.

One manifestation of poor inhibitory control is classroom distraction. Classroom learning is the most effective way for students to improve their academic performance, and if teachers fail to recognize that students’ attention has been diverted from the classroom during instruction, students’ academic performance does not improve even when they appreciate higher teacher support outside of class. In addition, in Chinese culture, teachers teach in large classes with approximately 40 students in each class, and these students have high and low levels of inhibitory control. Although teachers decide how fast to teach based on the cognitive level of the students, there are still students with poor inhibitory control who are unable to keep up with the teacher’s lectures, and this poor learning status can lead to poor academic performance.

For parents, BMI and academic achievement as a direct observation, overweight/obesity and low academic achievement are more easily perceived by parents, while inhibitory control as a cognitive ability that is not easily observed is easily ignored by parents, resulting in the inability to make targeted changes.

As mentioned above, poor inhibitory control in secondary school students may be due to the presence of peers with poor inhibitory control around them, and even though they give high social support, this social support does not correlate with inhibitory control and still does not improve the poor academic performance of inhibitory control secondary school students.

#### Educational Suggestions

Research has found that high BMI negatively affects academic achievement. This requires relevant educators to reverse the value bias of “emphasizing academic achievement over physical fitness” and to re-examine the positive role of physical fitness in cognitive ability and academic achievement by conducting lectures and regular studies. At the same time, schools should focus on strengthening students’ physical and mental health literacy education. From the cognitive point of view, students should scientifically estimate their BMI and clarify the dangers associated with overweight and obesity. On this basis, schools should take action to improve the comprehensive quality evaluation system for students, assess physical fitness, and include it in students’ academic performance.

The study found that the impaired inhibitory control of high BMI middle school students will lead to poor academic performance, which reveals that the inhibitory control and academic performance of overweight and obese adolescents can be effectively improved through intervention inhibitions, and schools, parents and individual students can improve inhibitory control through coordinated aerobic exercise and inhibitory control task training, and coordinated aerobic exercise includes physical activity in the form of cycling, basketball, rope skipping and other sports. These forms of physical activity can should be added to the student’s physical activity. Commonly used inhibitory control training tasks include Go/No-Go tasks, Stop Signal tasks, Stroop tasks, and Flanker tasks, which can be combined with video games, where students enjoy improving inhibitory control.

Teacher support, family support, and peer support were found to mitigate the negative effects of high BMI on academic performance. Teacher support and family support also mitigated the negative effects of high BMI on inhibitory control. Teachers should play a motivational role in teaching evaluation. Teachers should fully explore the strengths and potential of each student, especially overweight and obese students, give full recognition and motivation to the strengths, build students’ Self-Confidence, and give targeted help to the weaknesses. This requires that teachers must master the knowledge of educational psychology. Parents should give full play to their sense of responsibility. On the one hand, parents should learn about physical fitness and avoid underestimating their children’s weight. On the other hand, families should conduct regular meetings to ask about their children’s material and emotional needs and to create a harmonious and loving family atmosphere. Increasing peer acceptance, to make peer relationships harmonious, mental health teachers bear the main responsibility. Teachers promote listening and companionship among students by means of group counseling, such as forming mutual help groups and group game competitions, which highlights the need for mental health programs in schools.

### Limitations and Future Studies

First, this study adopted the convenient sampling method, selected only the first-year senior high school students in a middle school in Shanxi Province. The narrow age range of participants made the generalizability of the results of this study unknown, and further verification of the applicability of the findings to other samples is needed.

Second, the social support investigated in this study was the social support of all subject teachers, which did not take into account the correlation between teacher support and the corresponding academic achievement in different subjects and the measurement of social support by student Self-Report method, which is still somewhat different from objective teacher support.

Finally, this study hypothesized the relationship between BMI, inhibition control, and academic achievement based on previous studies and existing theories, and proved this relationship by collecting cross-sectional data, which would have been more convincing if a follow-up study method had been used.

Therefore, future research should consider expanding the types of teacher support to interdisciplinary and using a combination of observational methods, tracking studies, and standardized academic proficiency tests to explore in depth the effects of BMI, inhibitory control, and social support on academic achievement.

## Conclusion

This study investigated the effect of BMI on academic achievement and the role of inhibitory control and social support in it. Overweight obesity in secondary school students diminishes their inhibitory control and consequently reduces academic performance, while support from teachers, parents, and peers can ameliorate the negative effects of overweight obesity on academic performance, and teacher and parental support can also cushion the negative effects of overweight obesity on inhibitory control. Future studies need additional parameters (e.g., body fat percentage, GPA) to confirm our findings.

## Data Availability Statement

The datasets presented in this study can be found in online repositories. The names of the repository/repositories and accession number(s) can be found in the article/Supplementary Material.

## Ethics Statement

The studies involving human participants were reviewed and approved by The First Affiliated Hospital of Shihezi University, Shihezi University. Written informed consent to participate in this study was provided by the participants’ legal guardian/next of kin. Written informed consent was obtained from the individual(s), and minor(s)’ legal guardian/next of kin, for the publication of any potentially identifiable images or data included in this article.

## Author Contributions

YS designed the experiment, collected data, and prepared the manuscript. HY collected the data and made data analysis. SD corrected the whole language of the manuscript and made final approval. CM gave technique supports and valuable suggestions in experiment designing. All authors contributed to the article and approved the submitted version.

## Funding

This research was supported by the Xinjiang Graduate Scientific Research Innovation Project of China (XJ2021G092).

## Conflict of Interest

The authors declare that the research was conducted in the absence of any commercial or financial relationships that could be construed as a potential conflict of interest.

## Publisher’s Note

All claims expressed in this article are solely those of the authors and do not necessarily represent those of their affiliated organizations, or those of the publisher, the editors and the reviewers. Any product that may be evaluated in this article, or claim that may be made by its manufacturer, is not guaranteed or endorsed by the publisher.
